# Fluid Status Assessment in Critically Ill Patients with COVID-19: A Retrospective Cohort Study

**DOI:** 10.3390/jcm13020540

**Published:** 2024-01-18

**Authors:** Nadia Rodríguez-Moguel, Ivan Armando Osuna-Padilla, Karolina Bozena Piekarska, María-Fernanda Negrete-García, Andrea Hernández-Muñoz, Julián Andrés Contreras-Marín, Roberto Montaño-Mattar, Gustavo Casas-Aparicio

**Affiliations:** 1 Departamento de Investigación en Enfermedades Infecciosas, Instituto Nacional de Enfermedades Respiratorias “Ismael Cosío Villegas”, Mexico City 14080, Mexico; nadia.rodriguez@cieni.org.mx; 2 Departamento de Áreas Críticas, Instituto Nacional de Enfermedades Respiratorias “Ismael Cosío Villegas”, Mexico City 14080, Mexico; ivan.osuna@cieni.org.mx; 3 Departamento de Enseñanza, Instituto Nacional de Enfermedades Respiratorias “Ismael Cosío Villegas”, Mexico City 14080, Mexico; krola504@gmail.com (K.B.P.); fernanda.negf@gmail.com (M.-F.N.-G.); 05andrescontreras@gmail.com (J.A.C.-M.); robertomontanom77@gmail.com (R.M.-M.); 4 Facultad de Nutrición, Universidad Autónoma del Estado de Morelos, Cuernavaca 62209, Mexico; andrea.hernandez@uaem.edu.mx; 5 Departamento de Nefrología, Instituto Nacional de Enfermedades Respiratorias “Ismael Cosío Villegas”, Mexico City 14080, Mexico

**Keywords:** fluid overload, critically ill, COVID-19

## Abstract

Fluid status (FS) is a diagnostic challenge in critically ill patients with COVID-19. Here, we compared parameters related to FS derived from cumulative fluid balance (CFB), bioelectrical impedance analysis (BIA) and venous congestion assessed by ultrasound (VExUS) to predict mortality. We retrospectively reviewed the medical records of individuals with severe pneumonia due to COVID-19 between July and November 2021 in a single center. Comorbidities, demographic, clinical and laboratory data as well as results from CFB, BIA and VExUS measurements were collected on admission and weekly afterwards for two consecutive evaluations. Seventy-nine patients were included, of which eighteen (14.2%) died. Abnormalities of FS were only identified by BIA. Extracellular water/total body water ratio (ECW/TBW) > 0.394 (overhydrated) by BIA was a good predictor of mortality (AUC = 0.78, 95% CI: 0.067–0.89). Mortality risk was higher in overhydrated patients (OR: 6.2, 95% CI: 1.2–32.6, *p* = 0.02) and in persistently overhydrated patients (OR: 9.57, 95% CI: 1.18–77.5, *p* = 0.03) even after adjustment to age, serum albumin and acute kidney injury (AKI) in stages 2–3. Time to death was shorter in overhydrated patients (HR: 2.82, 95% CI: 1.05–7.5, log-rank test *p* = 0.03). Abnormalities in FS associated with mortality were only identified by BIA in critically ill patients with COVID-19.

## 1. Introduction

Fluid management is a crucial aspect of caring for patients with acute respiratory distress syndrome (ARDS), including those with coronavirus disease (COVID-19) [1]. ARDS is a severe respiratory condition characterized by widespread inflammation in the lungs, leading to difficulty in breathing and low oxygen levels [1,2,3]. Inappropriate fluid administration can exacerbate pulmonary edema, impair gas exchange and worse hypoxemia, making the lung stiffer and less compliant [4,5].

Critical care physicians conduct fluid balance primarily with a combination of clinical assessment, hemodynamic monitoring and laboratory values. However, the characteristics of patients with ARDS, particularly the diffuse and heterogeneous nature of interstitial inflammation and lung involvement, can limit the usefulness of certain diagnostic tools, including lung ultrasound and chest X-ray [6].

The lack of perception of cumulative fluid balance (CFB) and the inappropriate handling of intravenous solutions could lead to fluid overload (FO), increasing the risk of mortality [2,3]. The diagnosis of FO based only on CFB monitoring could be unrepresentative due to the pitfalls of the proper recording of fluid balance in intensive care units (ICU) [2,3]. The venous excess ultrasound (VExUS) grading system and bioelectrical impedance analysis (BIA) are methods for assessing systemic venous congestion and hydration status, respectively [7]. VExUS as a noninvasive method that evaluates venous congestion in the liver and kidneys could be a useful tool in guiding fluid management to avoid FO [8]. However, it may have limitations such as a lack of trained staff, interobserver variability, abdominal obesity and liver and heart diseases that need to be considered during the analysis of the results [8,9]. On the other hand, BIA assesses hydration status by analyzing the impedance of tissues to electrical currents, with multifrequency devices offering a more detailed picture of water compartments [10]. Considering that fluid accumulates primarily as extracellular water (ECW), overhydration can be calculated as the difference between expected extracellular water (ECW), based on the euvolemic extracellular water/total body water ratio (ECW/TBW), and the measured ECW [11]. The ECW/TBW ratio derived from BIA has been widely used to evaluate fluid status in multiple clinical conditions [12,13].

The aim of this study was to describe the longitudinal changes in fluid status and to identify which parameters derived from CFB, BIA and VExUS might be useful in predicting mortality in critically ill patients with COVID-19 and ARDS.

## 2. Materials and Methods

### 2.1. Study Population

This retrospective cohort study on critically ill patients with severe pneumonia due to SARS-CoV-2 was conducted between July and November 2021 at the National Institute of Respiratory Diseases in Mexico City, Mexico. Individuals over 18 years with SARS-CoV-2 confirmed by a positive real-time reverse-transcriptase polymerase chain reaction (rRT-PCR) test who were under invasive mechanical ventilation (IMV) and had undergone a weekly fluid status evaluation on the same day since their ICU admission were included. Patients with incomplete clinical records or poor acoustic window (confirmed by an ultrasound operator) and pregnant women were excluded. The Institutional Review Board (#C03-23) approved the study and waived the requirement for informed consent due to the retrospective design of the study. Data were fully anonymized before being accessed.

### 2.2. Fluid Status Evaluation in Critical Care Areas

Fluid status assessment was performed with BIA and VExUS as a routine plan in the ICU during the first 72 h after admission and then weekly during hospital stay. CFB as well as values from BIA and VExUS were collected.

#### 2.2.1. Body Composition and Hydration Assessment with Bioelectrical Impedance Analysis

Baseline body weight and height were estimated during the first 24–48 h after ICU admission using validated equations derived from anthropometric measurements. Body mass index (BMI) was calculated as BMI = body weight [kg]/height^2^[m] [14,15]. Body composition was assessed using a multifrequency device (InBody S10, InBody Co, Ltd., Seoul, Republic of Korea) with patients in a supine position. Estimated body weight and height were introduced into the device and eight adhesive electrodes were placed as follows: one on each wrist, one on the distal part of the third metacarpal bone of each hand, one on the central part of each ankle and one on the distal part of the second metatarsal bone in each foot. Extracellular water (ECW), intracellular water (ICW), total body water (TBW), extracellular water/total body water ratio (ECW/TBW) and intracellular water/total body water ratio (ICW/TBW) were obtained [10,11].

#### 2.2.2. Cumulative Fluid Balance (CFB) and Fluid Overload (FO)

Daily fluid balance was determined from all intakes and outputs recorded in monitoring charts. All available intake and output data since admission and every seven days during ICU stay were analyzed. We calculated fluid balance for each day using the sum of daily fluid intake (L) from which we subtracted total output (L). Total output included urinary volume and evacuations. To quantify CFB in relation to body weight, we used the following formula: (**Σ** daily (total fluid intake (L)-total output (L))/body weight (in kilograms)) [16]. Fluid overload (FO) was defined as >5% body weight gain between two measurements [3]. Insensible fluid loss is routinely a cause of concern when evaluating critically ill patients; because of this, we did not calculate insensible fluid loss [17].

### 2.3. Venous Congestion Assessed by Ultrasound Protocol (VExUS)

Ultrasound assessment was performed at bedside using a GE Venue ultrasound using a curvilinear abdominal probe of 1–5 MHz and a sectorial probe of 3 MHz by resident pulmonology physicians who had at least 2 years of experience in ultrasound examinations.

Patients were positioned in the dorsal decubitus position with the head end of the bed elevated at 0 grades. The inferior vena cava (IVC) was measured in a long-axis view, 1 cm after insertion of the hepatic vein (HV) in the subxiphoid position. Hepatic vein (HV) Doppler ultrasound was performed, visualizing one of the hepatic veins (left or middle) from the right subcostal view or the right hepatic vein from a lateral angle; a color flow doppler device was placed on the selected HV as it entered the IVC, and a pulse wave Doppler device was performed. Valuation of HV was made by identification and analysis of A, S and D waves: normal, S > D; moderate, S < D with antegrade S; severe, S flat or inverted or biphasic trace. A portal vein Doppler (PVD) assessment was obtained from a lateral approach, placing the probe in the right midaxillary line and placing over a color flow Doppler device, subsequently, a pulse wave Doppler device was placed over the portal vein. The interpretation of PVD was made by calculating the pulsatility index as (Vmax − Vmin)/Vmax) and interpreted as follows: normal, <0.3 pulsatility index; moderate, 0.3–0.49 pulsatility index; severe, >0.5 pulsatility index. A renal vein Doppler (RVD) assessment was also performed from a lateral view after placing the probe in the posterior axillary line, activating the color Doppler signal and the pulse wave Doppler signal over interlobar renal vessels. Intrarenal vein Doppler results was interpreted as follows: normal, continuous monophasic flow; moderate, biphasic flow with systolic/diastolic flow; severe, monophasic flow with only diastolic flow [8,9]. If respiratory liver movement prevented proper trace generation, the waveforms were traced during a respiratory pause.

The individual Doppler stages were compiled into VExUS staging of venous congestion as Grade 0 (IVC < 2 cm (cm)), Grade I (IVC of 2 cm or more with other flow patterns normal or with moderate pattern abnormalities), Grade II (IVC of 2 or more cm with one severe flow pattern abnormality) and Grade III (IVC of 2 or more cm with 2 severe abnormalities) [8,9].

### 2.4. Definition of Acute Kidney Injury (AKI)

Diagnosis and staging of AKI were based on the Kidney Disease Improving Global Outcomes (KDIGO) criteria, using only serum creatinine (sCr) levels. Baseline sCr level was defined as the minimum inpatient value during the first 7 days after admission [18].

### 2.5. Statistical Analysis

Normality in the distribution of quantitative variables was verified by the Shapiro–Wilk Test. Descriptive statistics were used to analyze categorical (absolute and relative frequency) and quantitative variables (mean and SD or median and interquartile range). Clinical data, CFB and variables derived from BIA and ultrasound evaluation were recorded and compared between survivors vs. non-survivors in each measurement using Student’s *t*-test, the Mann–Whitney U test or a chi-squared test. For each fluid status parameter (CFB, FO), BIA parameters (ECW/TBW, ICW/TBW) and VExUS parameters, the area under the receiver-operating characteristic curve (AUC) with 95% confidence intervals was calculated to predict mortality considering the baseline measurement. Sensitivity and specificity for the best cut-off value were determined.

The best cut-off value at the baseline measurement was taken to define overhydration. Clinical data, CFB, ICW/TBW ratio and VExUS variables between fluid-non-overhydrated vs. fluid-overhydrated patients were compared in each measurement using ANOVA for repeated measurements and a post hoc Bonferroni test. Univariate and multivariate Cox proportional hazard regression models were performed and adjusted to age, serum albumin <2.5 g/dL and AKI stages 2–3. Cumulative survival curves compared groups of overhydrated vs. non-overhydrated patients using the Kaplan–Meier method and were compared by the log-rank test. We considered a *p* value < 0.05 as significant. Data were analyzed using Stata Intercooled (version 14, StataCorp, College Station, TX, USA) and GraphPad Prism version 9.02 (GraphPad Software, Inc., San Diego, CA, USA).

## 3. Results

A total of 1176 individuals were admitted for evaluation at the emergency room during the study period. Of those, 786 individuals tested positive for SARS-CoV-2 infection, of which only 159 were admitted to the ICU. We included 79 patients that underwent the protocolized BIA and ultrasonographic evaluations. Sixty patients (75%) were men, the median age was 51.8 ± 16.4 years, twenty-two (29%) patients had systemic arterial hypertension, twenty (26%) had diabetes and twenty-three (29%) were obese. Baseline characteristics in survivors and non-survivor patients are shown in Table 1.

Baseline TBW was lower in the non-survivor group (36.9 ± 6.3 L vs. 41.7 ± 7.7 L in the survivor group (*p* = 0.019)) and lower at day 14 in the non-survivor group (35.2 ± 5.0 L vs. 41.4 ± 8.0 L in the survivor group (*p* = 0.044)); ECW/TBW ratio was higher at day 1 in the non-survivor group (0.39 ± 0.008 vs. 0.38 ± 0.01 in the survivor group (*p* = 0.004)) and higher at day 7 in the non-survivor group (0.40 ± 0.009 vs. 0.39 ± 0.01 in the survivor group (*p* = 0.020)) and at day 14 in the non-survivor group (0.40 ± 0.01 vs. 0.39 ± 0.01 in the survivor group (*p* = 0.002)). ICW/TBW was lower at day 1 in the non-survivor group (0.60 ± 0.008 vs. 0.61 ± 0.001 in the survivor group (*p* = 0.001)) and lower at day 7 in the non-survivor group (0.59 ± 0.009 vs. 0.60 ± 0.001 in the survivor group (*p* = 0.003)) and lower at day 14 in the non-survivor group (0.59 ± 0.009 vs. 0.60 ± 0.001 in the survivor group (*p* = 0.009)). There were no differences when analyzing CFB, fluid overload or VExUS parameters between the surviving vs. non-surviving groups (Table 2).

### 3.1. Performance of Mortality Predictors

Based on the highest AUC, specificity and accuracy values, the index with the best performance in predicting mortality was the baseline ECW/TBW ratio (AUC = 0.78, 95% CI: 0.067–0.89); the value 0.394 showed a sensitivity of 72% and a specificity of 68%; this cutoff was used to define overhydration. The AUC values of other predictors are shown in Table 3.

### 3.2. Survival Was Shorter in Overhydrated Patients

**Figure 1:**
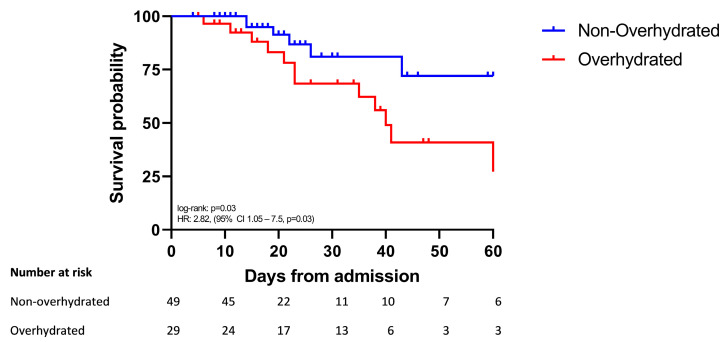
Kaplan–Meier survival curves. Time to death for the overhydrated group (blue line) vs. the non-overhydrated group (red line). Time 0 corresponded to ICU admission. Patients who were discharged alive before 60 days were treated as still at risk and not censored at discharge.

**Figure 2:**
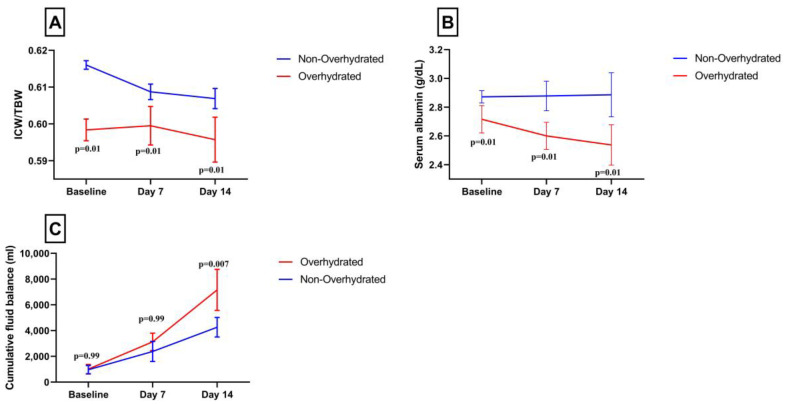
Illustration of the changes in ICW/TBW ratio (**A**), serum albumin (g/dL) (**B**) and cumulative fluid balance (mL) (**C**) at baseline, day 7 and day 14. The red line represents the overhydrated group and the blue line the non-overhydrated group. Data are presented as the mean and SEM. *p* < 0.05 for Bonferroni multiple comparison’s test, Abbreviations: ICW/TBW, ratio of intracellular water to total body water.

Twenty-nine patients (37%) were overhydrated (ECW/TBW > 0.394 ratio). Survival analysis showed that the time to death was shorter in overhydrated compared to the non-overhydrated patients (HR: 2.82, 95% CI: 1.05–7.5, log-rank test: *p* = 0.03) (Figure 1). Characteristics of ICW/TBW, serum albumin and CFB in each measurement in overhydrated vs. non-overhydrated patients are shown in Figure 2A–C.

### 3.3. Overhydration and Persistent Overhydration Were Risk Factors for Mortality

Logistic regression models indicated an increased risk in mortality in overhydrated and persistently overhydrated patients. The raw and adjusted models are shown in Table 4.

## 4. Discussion

The mortality rate among COVID-19 patients who required IMV due to ARDS is significantly increased [2]. Clinical data from patients with ARDS confirm that fluid overload has a deleterious impact on several outcomes at every phase of fluid therapy [19]. Given these potential adverse effects, it is crucial to properly guide fluid administration in patients with ARDS due to severe COVID-19 [2,19]. The aim of this study was to investigate the role of three different methods to assess volume status in critically ill patients with COVID-19 to predict mortality.

In this retrospective cohort of patients with severe COVID-19, we longitudinally analyzed the fluid status, evaluating the CFB, BIA and VExUS, from admission to critical care and then weekly for two consecutive measurements. In our research, the ECW/TBW ratio assessed by BIA was a good predictor of mortality with the cut-off value of >0.394 strongly associated with mortality, even after adjusting by age, serum albumin levels <2.5 g/dL and AKI at stages 2–3.

BIA, a valuable tool for assessing fluid status and body composition in various clinical settings, including critical ill patients, measures the opposition of tissues to an electrical current, and the results provide insights into different fluid compartments, including intracellular and extracellular water [13,20,21]. In critically ill patients, increased ECW/TBW has been linked to mortality, including patients with COVID-19 [7,22]. The ECW/TBW ratio reflects the proportion of extracellular water to total body water, and an elevated ECW/TBW ratio suggests an increased accumulation of extracellular water, which may indicate overhydration [23]. In our study, we observed, since early evaluations, changes in the composition of the ECW/TBW compartment, and we decided to define overhydration (ECW/TBW > 0.394) as a pathological expansion of the extracellular space that is associated with mortality and decreased survival. Overhydrated patients had significantly decreased ICW/TBW and low serum albumin levels in all measurements; all of these characteristics evaluated by BIA could reflect a phenotype that predisposes fluid sequestration to the interstitial space and that, as a whole, is associated with an increase in mortality. In line with our study, Park et al. were able to observe an immediate expansion of the extracellular compartment, assessed by BIA, after the early phase of fluid resuscitation; these changes were accompanied by a contraction of the intracellular space [12]. Susceptibility to early fluid sequestration in the extracellular compartment, which includes spaces such as interstitial areas, results in a syndrome known as interstitial edema. This pathological edema is strongly associated with mortality in critically ill patients [24].

In our cohort, we could not see differences in CFB or FO in any of the periodic evaluations between survivors and non-survivors. However, at day 14, the CFB was higher in overhydrated patients. This could indicate a late phase of fluid administration in our cohort; however, poor monitoring or incomplete documentation of fluid intake and output at early phases in the emergency room could lead to an inaccurate reflection of the fluid status in patients. Maintaining an accurate record of fluid balance is crucial in preventing complications related to overhydration [19,25]. However, while these measurements can provide valuable insights into fluid management, there are several factors that can introduce errors or inaccuracies into data collection and interpretation processes, such as a lack of standardized protocols for measuring and recording fluid intake and output, which can lead to inconsistencies in data collection [17].

In our study, we collected information from the VExUS protocol that was performed weekly as part of a fluid status assessment. However, we did not find a relationship between VExUS grade 2–3 and outcomes, such as mortality or AKI. Our results must be considered with caution because our study does not have the methodological rigor to evaluate these outcomes; likewise, we were not able to complete all three evaluations in all of the patients during follow-up, and this limits our study. The VExUS protocol is an emerging tool that has gained attention for its potential in evaluating venous congestion. The identification of venous flow patterns in abdominal organs using ultrasound imaging to identify venous congestion is the essence of the VExUS protocol; these changes are associated with adverse clinical outcomes [8]. A recent study in critical ill patients with sepsis and mechanical ventilation found a significant association between abnormalities in portal and hepatic vein Doppler signals and major adverse kidney events [26]; similar results have been observed in patients after cardiac surgery with abnormalities in portal vein flow [27,28]. In line with our study, a recent longitudinal study involving a cohort of critically ill patients with sepsis did not find an association between VExUS score at admission and AKI or mortality [29]. Given the still contradictory results, particularly in patients with sepsis, new studies with better prospective designs are currently being carried out with the aim of validating its usefulness in predicting outcomes such as the need for renal replacement therapy in critically ill patients with sepsis [30].

While our study did not evaluate other methods such as hemodynamic monitoring and passive leg elevation, the assessment of fluid status combining CFB, BIA and VExUS score provides a multifaceted view of fluid status in patients with severe ARDS and COVID-19. Incorporating dynamic assessments, such as passive leg elevation and hemodynamic monitoring, should be considered, especially in critically ill patients [31]. Passive leg elevation can provide insights into fluid responsiveness, and hemodynamic monitoring can offer real-time data on cardiac output, central venous pressure and other relevant parameters [32,33].

### Limitations

Our study has several limitations. Firstly, it is a retrospective observational study that is limited to a single center involving only the Mexican population with COVID-19. Secondly, considering the sample size of the present study, further studies are needed to corroborate our findings. Thirdly, although we were strict in obtaining fluid balance data from admission to intensive care, we could not check the accuracy of the registered data of the fluids that were administered in the emergency area and, therefore, we may not have accounted for the entire early phase of fluid administration and this probably influenced the fact that there was not an appropriate relationship between the increase in ECW/TBW and CFB of the first two measurements and, therefore, represents a bias. Lastly, all VExUS evaluations were made by trained pulmonologist fellows and we were unable to perform an interobserver variability analysis of the VExUS performed; in addition, a significant proportion of patients did not undergo the VExUS protocol in all three evaluations.

Finally, we consider that our study has the following strengths: Firstly, to our knowledge, there is no other study that has evaluated volume status with three different methods in this population of patients who have a high susceptibility to fluid overload. Secondly, although this was a retrospective study, we were able to collect objective weekly information on fluid balance, BIA and VExUS protocols.

## 5. Conclusions

Evaluation of fluid compartments in critically ill patients with COVID-19 is a complex process that requires repeated assessments due to the different clinical conditions that may lead to body fluid changes during ICU stay. Analysis of BIA together with other parameters could contribute to a better evaluation of fluid status in critically ill patients with COVID-19.

## Figures and Tables

**Table 1:** Demographic, biochemical and clinical data at baseline in survivors and non-survivors.

	Overall (*n* = 79)	Survivors (*n* = 61)	Non-Survivors (*n* = 18)	*p* Value
Age (years)	51.8 ± 16.4	48.1 ± 15.2	64.3 ±14.1	<0.001
Men %	60 (75)	48 (78.6)	12 (66.7)	0.290
Diabetes %	20 (26)	15 (24)	5 (18%)	0.870
Hypertension %	22 (29)	17 (28%)	5 (28%)	0.860
Obesity %	23 (29)	22 (36%)	1 (6%)	0.010
Laboratories				
Hemoglobin (g/dL)	13.6 ± 1.6	13.7 ± 1.5	13.3 ± 2.2	0.470
Creatinine (mg/dL)	0.91 (0.72–1.37)	0.9 (0.71–1.37)	0.98 (0.78–1.43)	0.360
BUN (mg/dL)	28 (21–39)	27.5 (18–36)	38 (24–54)	0.080
Sodium (mmol/L)	141 ± 5	141 ± 3.3	140 ± 9.2	0.620
Potassium (mmol/L)	4.4 ± 0.5	4.4 ± 0.5	4.5 ± 0.6	0.620
Phosphorus (mg/dL)	3.9 ± 1.0	3.9 ± 1.0	3.9 ± 1.0	0.770
Serum albumin (gr/dL)	2.8 ± 0.3	2.8 ± 0.3	2.7 ± 0.3	0.330
D-dimer (µg/mL)	0.9 (0.4–1.9)	0.9 (0.4–1.69)	1.5 (0.9–2.8)	0.055
Procalcitonin (ng/mL)	0.14 (0.08–0.46)	0.13 (0.07–0.34)	0.26 (0.2–0.46))	0.100
C-reactive protein (CRP) (mg/dL)	10.8 (4.3–15.8)	10.2 (3.8–15.6)	11.5 (9.3–16.4)	0.290
P/F ratio (PaO_2_/FiO_2_)	165 ± 45	168 ± 4	155 ± 47	0.320
AKI *n* (%)	29 (37%)	24 (39%)	5 (28%)	0.570
Hemodialysis (HD) *n* (%)	2 (3%)	1 (2%)	1 (5.5%)	0.320
Outcomes				
Hospital stay, Days	19 (14–35)	13 (8–25)	23 (14–40)	0.013
Invasive mechanical ventilation (IMV), Days	15 (9–27)	13 (8–25)	22 (14–35)	0.017

Abbreviations: BUN, blood urea nitrogen; CRP, C-reactive protein; PaO_2_/FiO_2_, relation of arterial blood oxygen pressure and fraction of inspired oxygen; AKI, acute kidney injury; HD, hemodialysis, AKI: acute kidney injury; IMV, invasive mechanical ventilation.

**Table 2:** Fluid status assessment in COVID-19 patients using fluid balance, BIA and VExUS.

	Survivors (*n* = 61)	Non-Survivors (*n* = 18)	*p* Value
Fluid balance			
CFB, baseline (mL)	840 (227–1402)	913 (−319–1898)	0.860
CFB mL/kg, baseline	10.4 (3.6–18.6)	9.5 (−6.0–23.3)	0.790
FO > 5%, baseline	2 (4%)	0	0.470
CFB, day 7 (mL)	2717 (1333–4577)	1852 (475–4389)	0.420
CFB mL/kg, day 7	27.3 (15.5–59.4)	26.9 (6.4–53.6)	0.510
FO > 5%, day 7	13 (21)	4 (22)	0.950
CFB, day 14 (mL)	6140 (1719–8944)	7131 (2920–16,995)	0.400
CFB mL/kg, day 14	67.8 (19.6–144.9)	124.3 (48.2–184.6)	0.250
FO > 5%, day 14	17 (28)	5 (28)	0.760
BIA			
TBW, baseline (L)	41.7 ± 7.7	36.9 ± 6.3	0.019
TBW, day 7 (L)	40.0 ± 8.8	36.2 ± 6.4	0.100
TBW, day 14 (L)	41.4 ± 8.0	35.2 ± 5.0	0.045
ECW/TBW, baseline	0.38 ± 0.01	0.39 ± 0.008	0.004
ECW/TBW, day 7	0.39 ±0.01	0.40 ± 0.009	0.020
ECW/TBW, day 14	0.39 ± 0.01	0.40 ± 0.01	0.002
ICW/TBW, baseline	0.61 ± 0.01	0.60 ± 0.008	0.001
ICW/TBW, day 7	0.60 ± 0.01	0.59 ± 0.009	0.003
ICW/TBW, day 14	0.60 ± 0.001	0.59 ± 0.009	0.009
VExUS			
VExUS 2–3 (%), baseline ^a^	11 (20%)	5 (36%)	0.410
VExUS 2–3 (%), day 7 ^b^	7 (18%)	3 (23%)	0.710
VExUS 2–3 (%), day 14 ^c^	4 (16%)	2 (28%)	0.460

^a^: Only 69 patients had VExUS scores: 55 survivors and 14 non-survivors; ^b^: Only 53 patients had VExUS scores: 40 survivors and 13 non-survivors; ^c^: Only 32 patients had VExUS scores: 25 survivors and 7 non-survivors. Abbreviations: BIA, bioimpedance analysis; VExUS, venous excess ultrasound score; CFB, cumulative fluid balance; FO, fluid overload; TBW, total body water; ECW/TBW, extracellular water/total body water; ICW, intracellular water; ICW/TBW, intracellular water/total body water.

**Table 3:** Area under the curve (AUC) to predict mortality according to day 1 indicators.

	AUC	CI 95%	Cut-Off Point	Sensitivity %	Specificity %
CFB mL/kg	0.47	0.28–0.66	>8	64%	43%
Fluid Overload (FO) > 5%	0.48	0.45–0.50	>1	0%	96%
VExUS 2–3	0.57	0.43–0.71	>1	36%	80%
ECW/TBW	0.78	0.67–0.89	0.394	72%	68%
ICW/TBW	0.21	0.10–0.32	0.595	56%	13%

Abbreviations: AUC, area under the curve; CI, confidence interval; CFB, cumulative fluid balance; VExUS, venous excess ultrasound score; ECW/TBW, extracellular water/total body water ratio; ICW/TBW, intracellular water/total body water ratio.

**Table 4:** Logistic regression models for mortality in overhydrated and persistently overhydrated patients.

	Overhydrated	Persistently Overhydrated
Model 1	5.0 (1.6–15.6, *p* < 0.01)	6.0 (1.4–25.1, *p* = 0.01)
Model 2	6.2 (1.2–30.6, *p* = 0.02)	7.6 (1.11–52.3, *p* = 0.03)
Model 3	6.2 (1.2–32.6, *p* = 0.02)	9.5 (1.18–77.5, *p* = 0.03)

Model 1. Crude; Model 2. Adjusted to age, low albumin (<2.5 g/dL); Model 3. Model 2 + AKI at stages 2–3.

## Data Availability

All data generated and analyzed during this study were included in a Supplementary Materials (S1 File Raw Data).

## Supplementary Materials

The following supporting information can be downloaded at: https://www.mdpi.com/article/10.3390/jcm13020540/s1.
